# A Novel Method of Detecting a Needle by Means of Magnetism, and Its Extraction

**Published:** 1847-01

**Authors:** R. T. Gill

**Affiliations:** New York


*A novel method of detecting a needle by means of Magnetism, and its extraction.* By R. T. Gill, of New York.—On Friday, November 13th, 1846, Miss D., while kneeling upon the carpet run a cambric needle into her knee, and broke it. The usual ineffectual search having been made, it occurred to me, that a magnetic needle would detect it, and if the needle could be charged, its poles might be located. For this purpose, the north pole of a horse-shoe magnet was drawn several times from above downwards, over the point of entrance. Then having charged a darning needle, suspended by means of a thread, and holding it near the point where the cambric needle had entered, it was found to have slight polarity.

The horse-shoe magnet was then bound below the knee, diagonally across, so as to present the north pole towards the point of entrance, that the needle might thus be charged more effectually by induction. On the 16th, a proper magnetic needle having been procured, and presented to the knee, its north pole was strongly attracted to a certain point, which was marked with ink ; then on presenting the south pole, and moving it up about three-quarters of an inch, it was strongly attracted, and that point also marked. An incision at right angles, bisecting the disc between the two marks, struck the needle at its centre. Having passed a curved needle under it, so as to fix it, then cutting down upon one of its points, it was extracted.

The needle had moved more than its length below the place of entrance, caused somewhat, possibly, by the attraction of the horse-shoe magnet.—*N. Y. Annalist.*

